# Origin and Demographic History of Philippine Pigs Inferred from Mitochondrial DNA

**DOI:** 10.3389/fgene.2021.823364

**Published:** 2022-01-25

**Authors:** John King N. Layos, Cyrill John P. Godinez, Lawrence M. Liao, Yoshio Yamamoto, Joseph S. Masangkay, Hideyuki Mannen, Masahide Nishibori

**Affiliations:** ^1^ Laboratory of Animal Genetics, Graduate School of Integrated Sciences for Life, Hiroshima University, Higashi-Hiroshima, Japan; ^2^ College of Agriculture and Forestry, Capiz State University, Mambusao, Philippines; ^3^ Department of Animal Science, Visayas State University, Baybay City, Philippines; ^4^ Laboratory of Aquatic Botany, Graduate School of Integrated Sciences for Life, Hiroshima University, Higashi-Hiroshima, Japan; ^5^ College of Veterinary Medicine, University of the Philippines, Los Baños, Philippines; ^6^ Laboratory of Animal Breeding and Genetics, Graduate School of Agricultural Science, Kobe University, Kobe, Japan

**Keywords:** demographic history, dispersal, migration, Philippine pigs, phylogeography, *Sus scrofa*

## Abstract

The Philippines is a mega-diverse country that lies at the crossroads of past human migrations in the Asia-Pacific region and is believed to have never been connected to the Asian continent, even during the major sea-level subsidence of the Quaternary. As a result, the history of pig dispersal in the Philippines remains controversial, due to limited molecular studies and absence of archaeological evidence of pig domestication. This study provides the first comprehensive analysis of 184 complete mitochondrial DNA D-loop region from Philippine pigs to elucidate their early dispersal history by performing a phylogenetic comparison with wild boars and domestic pigs worldwide. The results showed a demographic signal of the ancestry of Philippine pigs that had a close genetic relationship with those from the mainland Southeast Asia and Northeast Asia, suggesting gene flow that may have resulted from human migration and trade. Here we have suggested two possible dispersal routes. One parallels the Neolithic expansion in Island Southeast Asia and Oceania via Northeast Asia, the other from the mainland Southeast Asia, into Palawan and Sulu Archipelago as early as prehistoric times via the Sundaic Region. Despite geographic barriers to migration, numerous genetic lineages have persisted across the Philippine islands, even justifying the recognition of a Philippine Lanyu subclade. The prehistoric population history suggests a demographic expansion that coincided with the interglacial periods of the Pleistocene and may have spread from the southern regions into the eastern and central regions of the Philippines. The intriguing signal of discrepancy discovered between the ancestral pattern and distribution range of the numerous endemic Philippine wild pigs opens a challenging new approach to illuminate complexity among these animals. Our study has contributed significantly towards completing the sparse molecular studies on Philippine pigs, an essential for creating win-win conservation measures.

## Introduction

The Philippines is an archipelago of 7,641 islands situated in Island Southeast Asia (ISEA) tagged as a nexus of ancient human migrations within the western Pacific region (Arenas et al., 2020; Larena et al., 2021). It is a mega-biodiverse country with almost half of the terrestrial vertebrates and vascular plants considered endemic (Posa et al., 2008). Together with Madagascar it shares the distinction as both a mega-diverse country and a global hotspot for biodiversity conservation (Mittermeier et al., 1999). In recent decades, a growing community of biogeographers, population geneticists, conservation biologists, and phylogeneticists has begun to focus on the archipelago and its diverse, endemic life forms as a model system to investigate a variety of conceptual questions related to evolutionary diversification (Heaney et al., 2005; Brown and Diesmos, 2009; Brown et al., 2013; Oaks et al., 2013). Although the Philippines has always been characterized as a region of global priority for species conservation, the unforeseen threat of extinction of some animal genetic resources is well known (Heaney and Mittermeier, 1997; Myers et al., 2000; Roberts, 2002; Posa et al., 2008).

The Philippines has one of the highest wild pig diversities in the world. It harbors four endemic wild pigs such as the Philippine warty pigs (*Sus philippensis*), Visayan warty pigs (*Sus cebifrons*), Palawan bearded pig (*Sus ahoenobarbus*), and Mindoro warty pig (*Sus oliveri*), as well as one native shared with Sundaic biogeographic region, the Bornean bearded pig (*Sus barbatus*) (Oliver, 1995). Unfortunately, these wild species are listed as Critically Endangered in the International Union for Conservation Nature (IUCN) Red List (Oliver and Heaney, 2008). Although they do not receive much international attention, maintaining a viable population of these ecologically important species should be a high conservation priority.

It is interesting to note that the Philippines was never thought to be connected to the Asian continent, not even during the Quaternary sea-level subsidence (Voris, 2000). Thus, the faunal assemblages of the Philippines have become vital as it presents several palaeoecological, biogeographic, and archaeological questions and offers a unique evolutionary and ecological laboratory for understanding island biodiversity changes in Southeast Asia (Ochoa, 2019). For instance, the lack of archaeological evidence and molecular studies on *Sus scrofa* have poses a challenge in identifying its prehistoric arrival and domestication in the Philippines. The *S. scrofa* is a ubiquitous species that was not considered native to the Philippines and was likely introduced as a domestic animal within the last few thousand years (Ingicco et al., 2017). Studies have shown that *S. scrofa* has a very broad natural habitat and has been independently domesticated in different parts of the world (Groves, 1981; Giuffra et al., 2000; Larson et al., 2005) and has adapted to a variety of new environments in a relatively short evolutionary time frame (Frantz et al., 2016). A schematic profile of wild boar origin, dispersal and domestication across Eurasia has been well documented using mitochondrial DNA (mtDNA) from sequences of wild boar, domestic pigs, and ancient specimens worldwide (Bellwood and Dizon, 2005; Larson et al., 2005; Larson et al., 2007; Wu et al., 2007). In addition, long-term gene flow between domestic pigs and wild boars during and after domestication has been well documented throughout Eurasia (Giuffra et al., 2000; Kijas and Andersson, 2001; Franz et al., 2015; Yang et al., 2017). Although archaeological and genealogical evidence suggests that domestication of pigs occurred independently at multiple sites in Northeast Asia (NEA) and on the Mainland Southeast Asia (MSEA) (Wu et al., 2007; Larson et al., 2010; Yang et al., 2011; Jin et al., 2012; Li et al., 2017), and despite the role of the hypothesized Austronesian human expansion in ISEA (Bellwood and Dizon, 2005), the origin, dispersal, and domestication of pigs in the Philippines remain unclear. Thus far, the only potential domestic pigs identified in the archaeological record of the Philippines are from the Neolithic (4,000–3,000 cal. BP) and early Metal Age (3,000–2000 cal. BP) site at Nagsabaran in Northern Luzon, which confirmed the clear distinction between the domesticated pig and the Philippine warty pigs (Pipper et al., 2009; Amano et al., 2013), which is associated with the Neolithic expansion into ISEA and Oceania by Austronesian-speaking populations (Larson et al., 2005). However, this has recently been questioned as there is no evidence of domestic pigs in Taiwan at a similarly early date, casting doubt on the possible Neolithic introduction of domestic pigs to the Philippines (Li et al., 2015).

The only leading theory postulates that the Philippine pig is a product of indiscriminate interbreeding between numerous domesticated endemic Philippine wild pigs and an introduced pig breed (Eusebio, 1969) that was able to survive and reproduce even with minimal human intervention. However, this hypothesis remains tentative due to the paucity of molecular studies to support this claim, as the evolution and dispersal of Philippine pigs have yet to be elucidated. Today, they are very common even in the remotest villages throughout the country. Since they are among the indigenous animals found in most rural agricultural areas, they are of great importance for supplementary income, high quality protein food, and socio-cultural and economic services, especially in cultural festivals and ceremonies. Therefore, genetic studies of the diversity of these ecologically and economically important animals should be a priority for conservation strategies, as they represent excellent genetic resources for local economies and could also serve as a genetic basis for studying human settlement and migration.

On the other hand, mtDNA is a very informative genetic marker to study genetic diversity, relationships, and variability within and between populations (Giuffra et al., 2000; Kijas and Andersson, 2001; Yue et al., 2016; Ming et al., 2017; Arenas et al., 2020). Studies using the mtDNA variation has been effective in establishing the relationships between domestic species and wild relatives (Bruford et al., 2003), identifying domestication sites (Larson et al., 2005; Naderi et al., 2007; Larson et al., 2010), and tracing the maternal origin of the population back to ancient times (Upadhyay et al., 2017; Margeta and Margeta, 2019). Meanwhile, the displacement loop (D-loop) region of the mtDNA tends to be widely used because of its higher variation than the remaining regions of the mitogenome (Cann et al., 1984; Wang et al., 2019) and thus, has been frequently used for phylogenetic studies of closely related groups, especially for determining intra-specific phylogenies (Seabusry et al., 2011). In the present study, we aim to determine the genetic diversity, phylogeography, population dynamics, and extent of genetic introgression of Philippine pigs using the mtDNA D-loop region and to contribute important insights towards elucidating the history of pig dispersal and evolution worldwide.

## Materials and Methods

### Sample Collection and Ethical Approval

**FIGURE 1:**
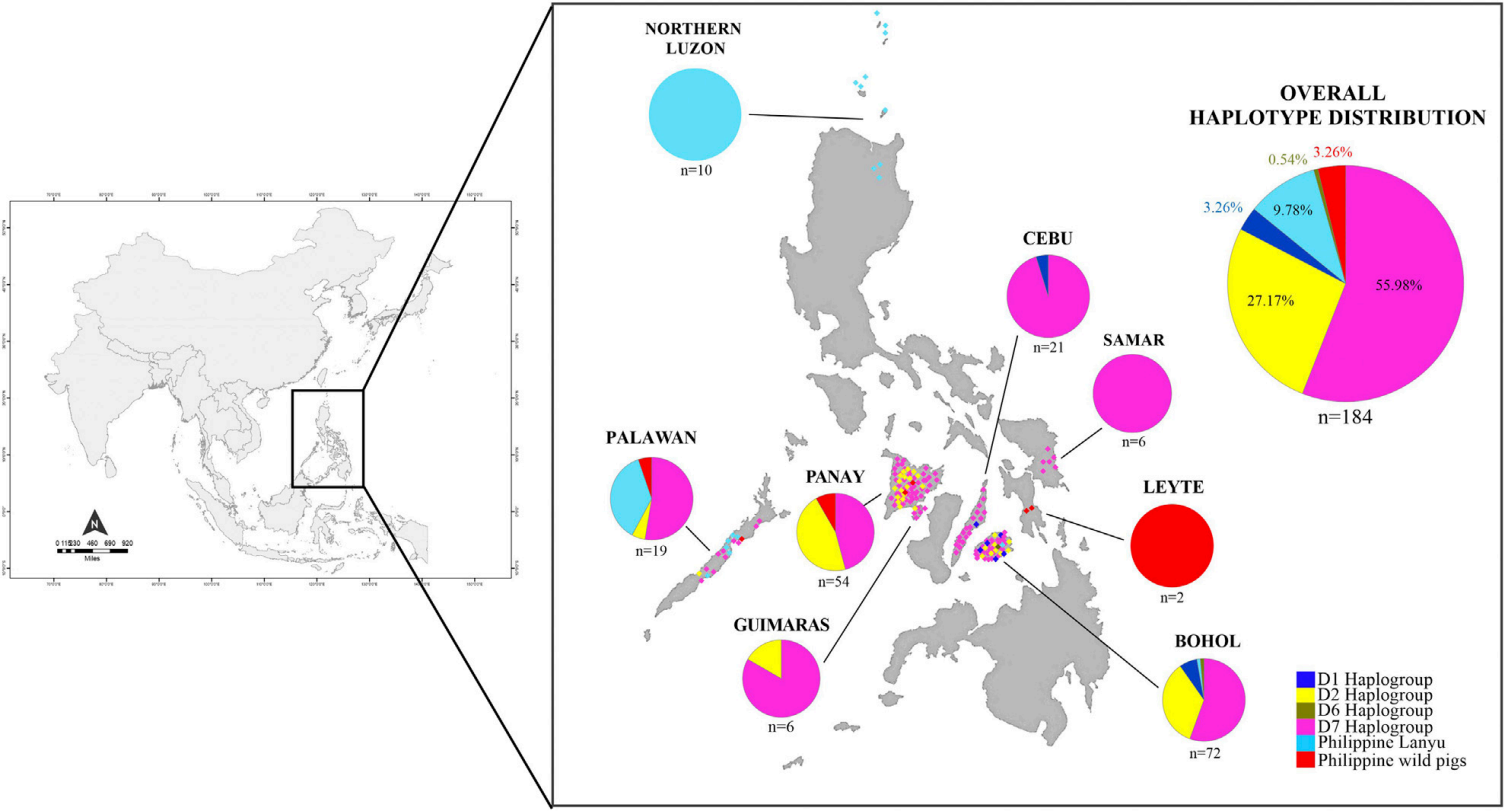
Distribution map of Philippine pigs and ancestry coefficients visualizing the geographic distribution of the different haplogroups found in this study.

**FIGURE 2:**
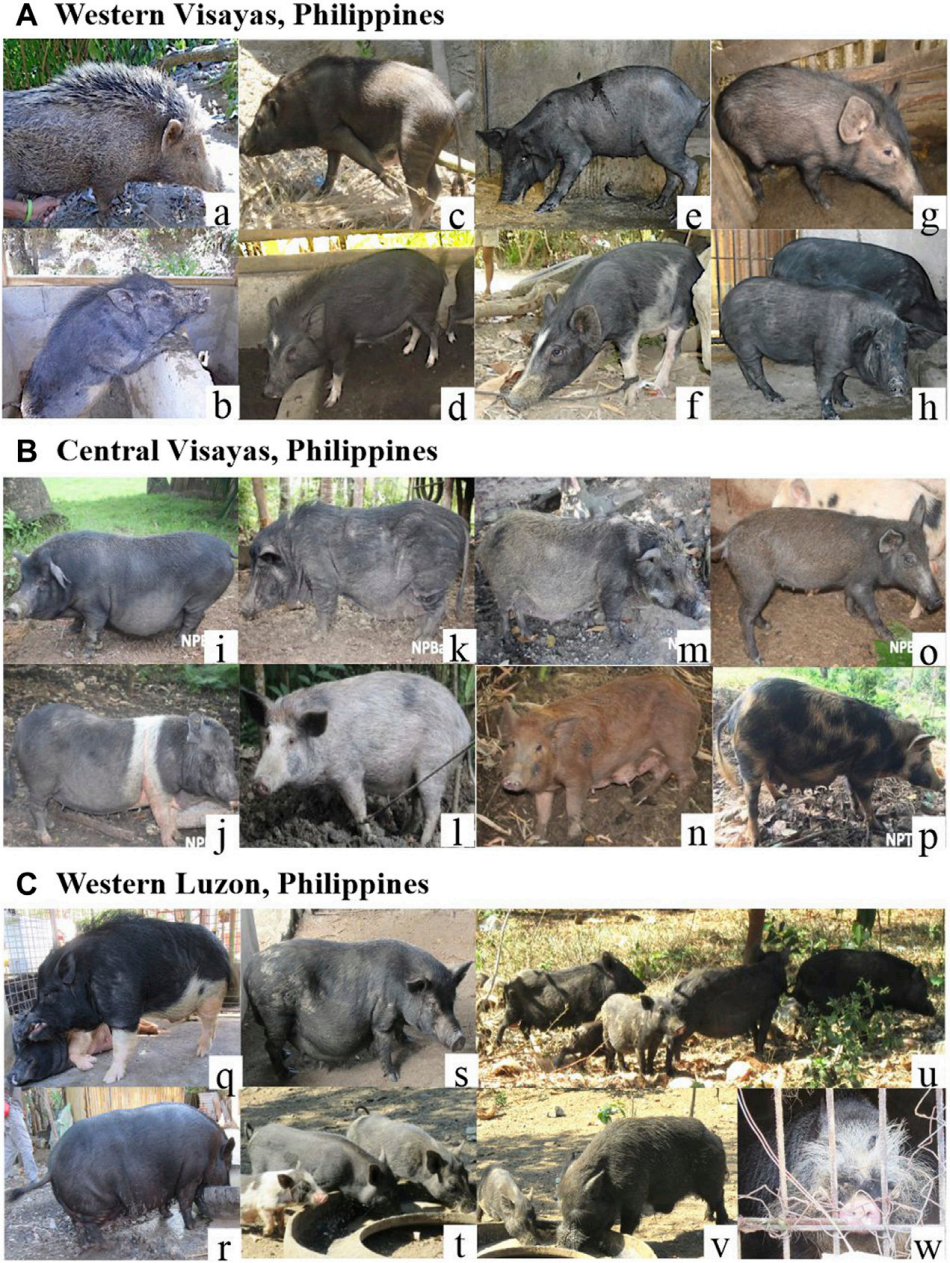
Photographs of Philippine pigs across the Philippine islands and their raising system. **(A)**: **(a)** Bugasong, Antique; **(b)** Balderama, Antique; **(c)** Banga, Aklan; **(d)** Numancia, Aklan; **(e)** Dingle, Iloilo; **(f)** Conception, Iloilo; **(g)** Mambusao, Capiz; **(h)** Sapian, Capiz. **(B)**: **(i)** Balilihan, Bohol; **(j)** Bilar, Bohol; **(k)** Balilihan, Bohol; **(l,m,n)** Guindulman, Bohol; **(o)** Talibon, Bohol; **(*p*)** San Miguel, Bohol; **(C)**: **(q)** Irawan Village, Puerto Princesa, Palawan; **(r)** Sandoval Village, Narra, Palawan; **(s)** Puerto Princesa, Palawan; **(t,u)** Dumarao Village, Roxas, Palawan; **(v)** Sandoval Village, Narra, Palawan (crossed endemic *Sus ahoenobarbus* with native pigs); **(w)** *Sus ahoenobarbus* (a wild pig endemic to Palawan Faunal Region).

Our experimental procedures were conducted in accordance with institutional and national guidelines governing the care and use of animals in experiments as established by the Laboratory of Animal Genetics, Hiroshima University (No. 015A170426). A total of 184 samples consisting of 175 Philippine native pigs (PHnp), six Philippine wild pigs (PHwp), and three crossbred PHnp to PHwp (part of the governments breeding and conservation program) were collected from 2017 to 2019 from nine localities in Central Visayas (*n* = 93), Western Visayas (*n* = 54), Western Luzon (*n* = 19), Eastern Visayas (*n* = 8), and 10 downloaded GenBank sequences from Northern Luzon (Figure 1; Supplementary Table S1). Since most of our sampling was done in the remotest areas across the Philippines, the absence of pedigree records was one of the limitations in this study. Therefore, the owners were interviewed to ascertain the unrelatedness of our samples and the Guidelines of Measurements of Domestic Animals Diversity Program set by the Food and Agriculture Organization (FAO, 2011) were strictly implemented throughout the sampling procedure. Photographs were taken to document the morphological characteristics and differences within these pig populations (Figure 2). The owners of the animals were personally consented to have their animals included in this study.

### DNA Extraction, PCR Amplification and Sequencing

Genomic DNA was extracted from whole blood and meat tissues of Philippine pigs using the phenol-chloroform method according to the recommended protocol of Green et al. (2012). For hair samples, the ISOHAIR kit (available at http://www.nippongene.com) was used for DNA isolation.

The 5.0-kbp of mtDNA fragment was first amplified with a Long and Accurate-PCR (LA -PCR) kit (KOD FX- Neo polymerase, TOYOBO, Otsu, Japan) using the established primer set, forward: *Sus* mt. 5.0 FL −2: 5′-ATG​AAA​AAT​CAT​CGT​TGT​ACT​TCA​ACT​ACA​AGA​AC-3'; reverse: Mum R: 5′-TTC​AGA​CCG​ACC​GGA​GCA​ATC​CAG​GTC​GGT​TTC​TAT​CTA-3'. The reaction began with an initial denaturation at 94°C for 2 min, followed by 30 cycles of denaturation at 98°C for 10 s, annealing at gradients at 57°C for 30 s, and primer extension at 68°C for 2 min and 30 s. The last step was a final extension at 68°C for 8 min. For complete amplification of the mtDNA displacement (D-loop) region, approximately 1.3 kbp fragment (15,434–16,679 sequenced positions of the mitogenome) was amplified with another primer set, forward: *Sus* mtD F1: AAC​TCC​ACC​ATC​AGC​ACC​CAA​AG; reverse: *Sus* mtD R1: CAT​TTT​CAG​TGC​CTT​GCT​TTG​ATA. The PCR reaction was performed in a total volume of 20 μl and the final concentrations of each component were as follows: 4.5 μl ddH2O, 10 μl 2× buffer, 4.0 μmol/L dNTPs, 0.3 μl of each primer (10 Pmol) (F & R primer), 0.6 μL KOD-FX Neo, and 0.5 μl genomic DNA. The reaction began with an initial denaturation at 94°C for 2 min, followed by 30 cycles of denaturation at 98°C for 10 s, annealing at gradient 59°C for 30 s, and extension at 68°C for 30 s. The last step was a final extension at 68°C for 5 min. Amplification was performed using GeneAmp PCR System 9,700 (Applied Biosystems, Foster City, CA, United States). The PCR products from the segmental amplification were purified with Exonuclease I (ExoI) and Shrimp Alkaline Phosphatase (SAP) to degrade the remaining PCR primers and dephosphorylate the remaining dNTPs, respectively. Then, the mtDNA D-loop fragments were sequenced with 3,130/3130xl Genetic Analyzers (Applied Biosystems, Foster City, CA, United States).

### DNA Sequence Alignment

The complete sequences of the mtDNA D-loop were assembled from the overlapping forward and reverse sequences using GeneStudio™ Professional, available at http://www.genestudio.com. Profile alignments of the sequenced data were performed using the ClustalW algorithm (Thompson et al., 1994) as implemented in Molecular Evolutionary Genetics Analysis (MEGA) (Tamura et al., 2013) to generate refined and continuous sequences for each animal. The nucleotide sequences were evaluated relative to the representative haplotypes of Asian domestic pigs under accession number AB041480 (Supplementary Table S1) along with the complete worldwide sequences of domestic and wild boars (Supplementary Table S2). About 1,044 bp of the complete mtDNA D-loop sequences were aligned and edited until one tandem repeat motif (5′-CGTGCGTACA-3′) remained, as the number of repeats was variable within individuals, indicating a high degree of heteroplasmy (Ghivizzani et al., 1993), and thus the repeat itself is not phylogenetically informative. The haplotype sequences were submitted to the GenBank National Center for Biotechnology Information (NCBI) databases with accession number OL957183-OL957251, MN625805-MN625830 and MW924902-MW92973.

### Genetic Diversity and Phylogenetic Reconstruction

The diversity measures such as the number of polymorphic segregating sites, haplotype diversity, and nucleotide diversity were estimated using DNA Sequence Polymorphism (DnaSP) 5.10 software (Librado and Rozas, 2009).

Two datasets were assembled for the phylogenetic analyses. The first dataset was the newly sequenced data from 184 animals used to study the genetic structure of the Philippine pig population. In the second dataset, we downloaded complete mtDNA D-loop sequences of global domestic and wild boars representing Asian and European pigs from GenBank to make further inferences about the relationships and demographic distribution of the Philippine pig populations (Supplementary Table S2). The Bayesian phylogenetic tree based on posterior probabilities was constructed using the program MrBayes 3.2 (Ronquist et al., 2012), using HKY + G + I as the best-fitted model of molecular evolution determined using MEGA 7.0.26 (Tamura et al., 2013) and jModelTest based on the Bayesian information criterion (Darriba et al., 2012). Trees were rooted with Warthog (*Phacochoerus africanus*; DQ409327). For each tree, two independent Marcov chain Monte Carlo (MCMC) were run for 2 × 10^7^ (first dataset) and 5 × 10^7^ chain length (second dataset), sampled every 1,000 generations. The first 10% of the sampled trees and estimated parameters of each dataset were discarded as burn-in. To obtain sufficient convergence of log-likelihood values, a standard deviation of < 0.05 was considered. The phylogenetic tree using maximum likelihood (ML) was also constructed. The phylogenetic consensus of Philippine wild pigs was constructed using the ML algorithm method with the model of GTR + R implemented in PhyML v.3.0. (Guindon et al., 2010). The consensus trees were illustrated using FigTree 1.3.1. Using BioEdit ver.7.1 (Hall, 1999), we further examined the haplogroup-specific mutations in all our samples to justify the haplogroup assignment of each sequence.

### Haplogroup and Geographic Classification

To obtain more detailed information about the genealogical relationship between haplotypes, we constructed a median-joining (MJ) networks (Bandelt et al., 1999) using PopArt 1.7 (Leigh and Bryan, 2015). This method calculates the net divergence of each taxon from all other taxa as the sum of the individual distances from variance within and among groups. The nomenclatures described by Larson et al. (2005) with six clades (D1 to D6) including the newly proposed mitochondrial Southeast Asia (MTSEA) haplogroup (Tanaka et al., 2008), previously renamed D7 by Layos et al. (2021), were used as a reference for clade notation. We also performed the network analysis on shorter sequences (509 bp) to accommodate the major representative haplotypes by partial mtDNA resolution, which were used in the previous studies for a thorough haplotype representation of the different haplogroups and geographical locations.

### Population Expansion Estimation and Demographic History Analysis

Deviations from selective neutrality were estimated using Fu (1997) F_*S*_ based on a coalescent simulation algorithm and Tajima’s *D* statistical tests using Arlequin (Excoffier et al., 2005), and their significance was tested over 1,000 coalescent simulations. The Fu’s F_*S*_ test is very sensitive to demographic expansion, resulting in large negative F_*S*_ values, whereas the significant Tajima’s *D* value could be a sign of population expansion and bottleneck (Tajima, 1989).

The past population dynamics were examined with the Bayesian Skyline Plot (BSP) model (Drummond et al., 2005) with standard MCMC sampling procedures under HKY + G model of substitution (Hasegawa et al., 1985) with four gamma categories using BEAST v.2.6.3 (Bouckaert et al., 2014). The BSP represents changes in population size over time derived from mtDNA and assumed mutation rate. Analyses were performed for the entire dataset and the predominant haplogroups D2 and D7 using a mutation rate of 1.36 × 10^−8^ (mutation rate per nucleotide site per year according to previous estimates for the D-loop of mammalian mtDNA; Pesole et al. (1999)) using the strict molecular clock model. MCMC analysis was performed for 5×10^7^ generations. Independent runs (logs and trees) were pooled using Log Combiner, discarding the first 10% burn-in and sampling parameter values every 5,000 generations. We ran the MCMC simulation twice independently for all datasets to ensure that the simulation converged at the same rate. Tracer v.1.7 (Rambaut et al., 2018) was used to confirm the correct convergence of the MCMC chain with an effective sample size (ESS) > 200 in the log files and to visualize the dynamics of the effective population size over time. The light blue shaded area in Figure 6 marks the 95% highest posterior density (HPD). The *X*-axes are time in thousands of years before present (BP) and the *Y*-axes are mean effective population size (*N*_*e*_) in millions of individuals divided by generation time on a logarithmic scale.

## Results

### Mitochondrial DNA Variation and Genetic Diversity

Among the 184 sequences, we identified 49 haplotypes (PHL1-PHL49), 25 of which were found only once among the sequences (Table 1). Of the 25 private haplotypes, eight were from Bohol, seven from Western Visayas, six from Palawan, three from Samar, and one from Cebu. The geographic distribution of these haplotypes is shown in Figure 1. When the distribution of these 49 haplotypes is summarized, 45 haplotypes occurred in PHnp and four are unique to PHwp. PHL1 was the most common haplotype, shared by 43 individuals (24.37%) and had the largest geographic distribution across all sampling sites except Northern Luzon. To avoid overestimating the expected values of the genetic diversity indices, we did not include the PHwp haplotypes in the calculation due to the high genetic variation in the sequences. In the 45 PHnp haplotypes, we detected 69 polymorphic sites delineated by 55 transitions and 14 transversion sites. The distribution of nucleotide positions and sequence variations of the haplotypes are shown in Supplementary Table S1. The overall diversity of haplotypes was 0.968 ± 0.004 and ranged from 0.889 ± 0.019 (Bohol) to 0.556 ± 0.075 (Northern Luzon). Total nucleotide diversity was 0.009 ± 0.005 and ranged from 0.0134 ± 0.004 (Palawan) to 0.0005 ± 0.001 (Northern Luzon) (Table 2).

**TABLE 1:** List of haplotypes and their geographic distribution.

Haplotypes	Geographic Distribution							Total
	Bohol	WesternVisayas	Cebu	Samar	Palawan	Leyte	Northern Luzon	
PHL1	17	13	5	0	8	0	0	43
PHL2	7	8	2	0	1	0	0	18
PHL3	6	5	0	0	0	0	0	11
PHL4	7	0	1	0	0	0	0	8
PHL5	1	0	0	0	0	0	0	1
PHL6	1	0	0	0	0	0	0	1
PHL7	10	0	0	0	0	0	0	10
PHL8	1	0	0	0	0	0	0	1
PHL9	1	0	0	0	0	0	0	1
PHL10	1	0	0	0	0	0	0	1
PHL11	1	0	0	0	0	0	0	1
PHL12	2	0	0	0	0	0	0	2
PHL13	1	0	0	0	4	0	0	5
PHL14	4	0	0	0	0	0	0	4
PHL15	4	0	0	0	0	0	0	4
PHL16	2	0	0	0	0	0	0	2
PHL17	4	0	1	0	0	0	0	5
PHL18	1	0	0	0	0	0	0	1
PHL19	1	0	0	0	0	0	0	1
PHL20	0	0	0	0	1	0	0	1
PHL21	0	0	0	0	1	0	0	1
PHL22	0	0	0	0	1	0	0	1
PHL23	0	0	0	0	1	0	0	1
PHL24	0	0	0	0	1	0	0	1
PHL25	0	8	0	0	0	0	0	8
PHL26	0	2	0	0	0	0	0	2
PHL27	0	1	0	0	0	0	0	1
PHL28	0	1	0	0	0	0	0	1
PHL29	0	2	0	0	0	0	0	2
PHL30	0	1	0	0	0	0	0	1
PHL31	0	2	0	0	0	0	0	2
PHL32	0	1	0	0	0	0	0	1
PHL33	0	1	0	0	0	0	0	1
PHL34	0	3	0	0	0	0	0	3
PHL35	0	2	0	0	0	0	0	2
PHL36	0	0	0	3	0	0	0	3
PHL37	0	0	0	1	0	0	0	1
PHL38	0	0	0	1	0	0	0	1
PHL39	0	0	0	1	0	0	0	1
PHL40	0	0	6	0	0	0	0	6
PHL41	0	0	1	0	0	0	0	1
PHL42	0	0	2	0	0	0	0	2
PHL43	0	0	3	0	0	0	0	3
PHL44	0	1	0	0	0	0	0	1
PHL45	0	0	0	0	0	2	0	2
PHL46	0	1	0	0	0	0	0	1
PHL47	0	1	0	0	1	0	0	2
PHL48	0	0	0	0	0	0	5	5
PHL49	0	0	0	0	0	0	5	5
TOTAL	72	54	21	6	19	2	10	184

**TABLE 2:** Genetic diversity indices and values of neutrality test statistics of Philippine pigs including the predominant D2 and D7 haplogroups.

					Neutrality Test	
Location	*n*	*h*	Haplotype Diversity	Nucleotide Diversity	Tajima’s *D*	Fu’s F_*S*_
Bohol	72	19	0.899 (0.019)	0.0072 (0.0010)	−0.56236	−0.18375
Palawan	18	8	0.778 (0.086)	0.0134 (0.0041)	0.93285	4.44524
Western Visayas	51	14	0.872 (0.028)	0.0055 (0.0029)	−0.74446	0.46914
Samar	6	4	0.800 (0.172)	0.0018 (0.0014)	−0.82582	−0.62499
Cebu	21	8	0.857 (0.047)	0.0054 (0.0027)	−0.96728	1.31326
Northern Luzon	10	2	0.556 (0.075)	0.0005 (0.0005)	—	—
OVERALL	178	46	0.968 (0.004)	0.0091 (0.0046)	−0.5564*	−16.3625**
D2	49	16	0.884 (0.024)	0.0044 (0.0024)	−1.1142*	−2.3181*
D7	103	20	0.784 (0.035)	0.0029 (0.0017)	−0.6570*	−5.9658**

n = number of samples; h = number of haplotypes; *p < 0.05; **p < 0.01 as tested by randomization (1,023 permutations) using Arlequin.

### Phylogeography and Distribution of Philippine Pig Haplogroups

For the first dataset, the 49 haplotypes were used to infer the population genetic structure of Philippine pigs based on the complete mtDNA D-loop sequences (1,044 bp). The phylogenetic tree generated from the Bayesian tree showed clear maternal-genetic divergence in the Philippine pig populations and revealed eight topologies of phylogenetically distinct clades with posterior probabilities ranging from 72 to 100% (Supplementary Figure S1). The two clades in the basal position of the phylogenetic tree that represented the Philippine wild pig samples with significantly high posterior probabilities fell outside the *S. scrofa* lineages.

**FIGURE 3:**
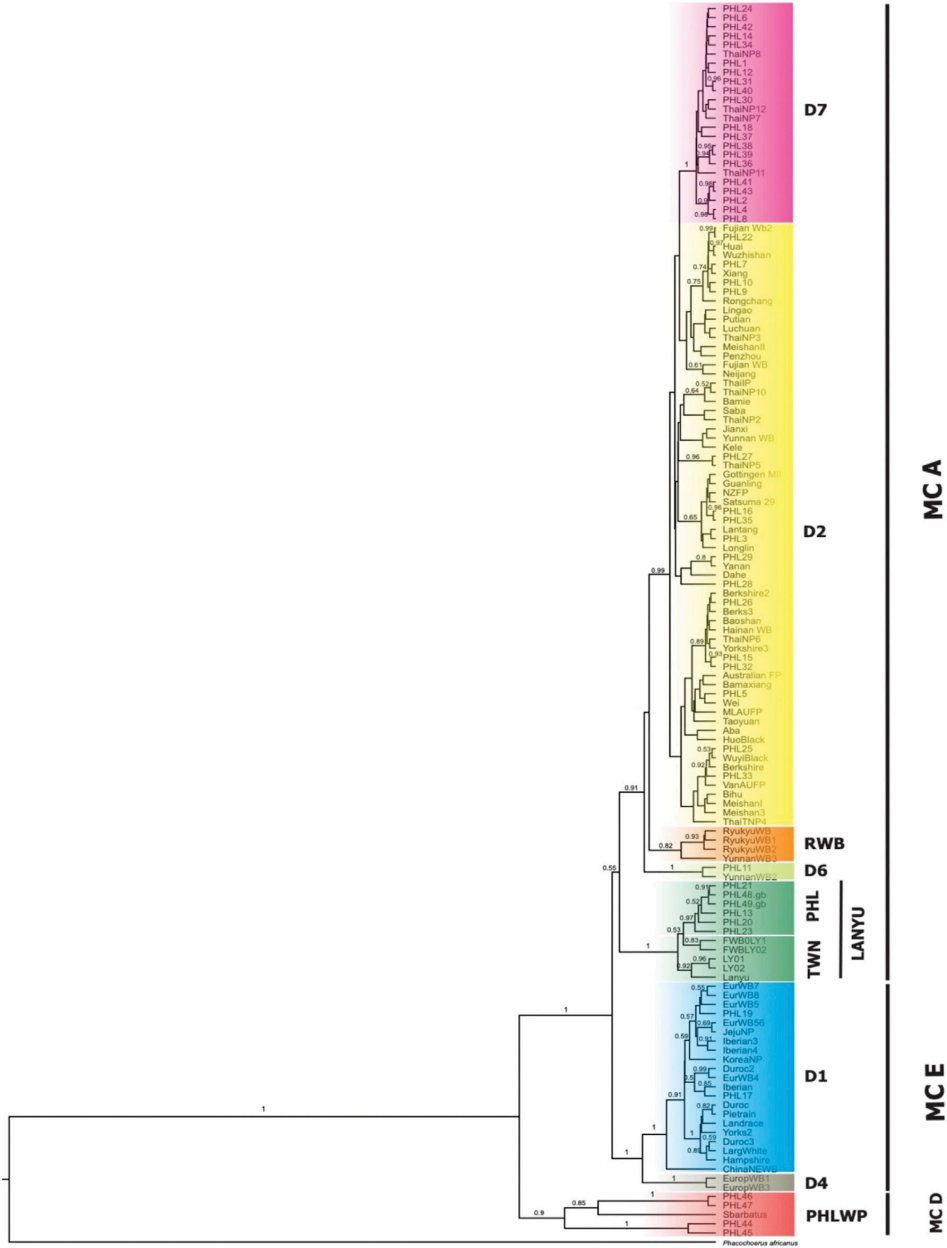
Bayesian tree inference with HKY + G + I as the best-fitted model using MrBayes 3.2 with Warthog (*Phacochoerus africanus*) as the outgroup. All Philippine pig haplotypes (PHL1-49) were combined with the downloaded sequences from GenBank classified as domestic and wild *Sus scrofa* corresponding to their geographic origin. Macro-clades (MC) A and E represent Asian and European pigs, respectively, forming further subclades. The MC D was designated for Philippine pig haplotypes that were outside the *Sus scrofa* lineages.

For the second dataset, we performed the phylogenetic analysis using both Bayesian and ML tree inference. The phylogenetic tree, both Bayesian (Figure 3) and ML (Supplementary Figure S2), showed a fundamentally similar topology, revealing two macro-clades (MC), which we designated A and E, while D represented samples that formed outside the wild *S. scrofa* lineages. Macro-clades A and E were represented by domestic pigs and wild boars that intermingled, one having an Asian and the other of European phylogeographic origin. The MC D represents the Philippine wild pigs (*n* = 6/184; 3.26%), which formed a unique lineage that fell outside the MCs A and E with significant posterior probability and was distinct from the *S. scrofa* lineages. Forty of the 49 haplotypes of the Philippine pigs (*n* = 172/184; 93.48%) were randomly arranged in MC A and formed further sub-clades, while MC E assembled the European wild boars and exotic domestic pigs with Northeast Asian domestic and wild boars together with the Philippine pigs (*n* = 6/184; 3.26%).

By screening and detecting the haplogroup-specific mutational motifs, we assigned each haplotype based on the smallest named sub-phylo groups to which it belongs. Recently, it has been proposed that the six major clusters of porcine mtDNA sequences, designated D1 to D6 (Larson et al., 2005), with the addition of D7 (previously designated MTSEA, which is thought to be restricted to the Indo-Burma Biodiversity Hotspots (IBBH); Tanaka et al., 2008; Larson et al., 2010; Layos et al., 2021) reflect domestication from genetically distinct subpopulations of wild boars (Supplementary Table S3). Our results showed that the D-loop sequences of Philippine pigs obtained in this study could be classified into five phylogenetically distinct haplogroups such as D1, D2, D6, D7, and Lanyu Clade, except for the Philippine wild pig haplotypes (PHL44-47). At MC A, 16 out of 49 haplotypes (*n* = 49/184; 27.17%) formed a paraphyletic clade together with the various domestic and wild boars distributed in the NEA region. This haplogroup was previously classified as the D2 haplogroup, which included most of the major Asian domestic and wild boars, corresponding to the widely distributed Chinese domestic pigs, a worldwide pig breed that has some relationship with Asian pigs, and the East Asian wild boars (Okumura et al., 2001; Fang and Andersson, 2006; Larson et al., 2010). In this haplogroup, there were four haplotypes such as PHL3 (11 individuals), which showed genetic relatedness to Gottingen, Lantang, and Satsuma, a domestic pig found in Germany, China, and Japan, respectively; PHL7 (10 individuals) with Xiang pig; PHL25 (7 individuals) with Wuyi black pig; and PHL26 (2 individuals) with Hainan wild boar from South China, along with modern western pigs such as the Berkshire and Yorkshire lineages, that have Asian matrilineal ancestry. Haplogroup D2 is widely distributed in Bohol Island and in all five provinces of Western Visayas such as Capiz, Iloilo, Aklan, Antique and Guimaras. Thus, the placement of these haplotypes in the phylogeny is not composite, suggesting a derived ancestral population from the numerous wild boars and domestic pigs from the NEA region.

Based on the patterns of mutational signatures, the most numerous haplotypes (20 haplotypes; *n* = 103/184, 55.98%) in our dataset which covered the largest area, formed an independent clade although this haplogroup was not present in Northern Luzon. While this study is the first analysis to resolve the complete mtDNA D-loop of a substantial number of sequences from these populations, it shows that it has an analogous signature to the previously documented haplogroup in MSEA, which was classified as D7 based on short/partial fragment analysis (510 bp) (Tanaka et al., 2008; Layos et al., 2021). Using complete D-loop fragment analysis, we confirmed that this haplogroup is distinct from the available haplogroup that has been tentatively classified by haplogroup-specific motif recognition in porcine mtDNA (Larson et al., 2005; Wu et al., 2007). The Philippine pig haplotypes in this haplogroup harbored two unique mutational derivatives at positions G24A and A893G that were not detected in other sequences from wild boars and domestic pigs worldwide, except for the three haplotypes from Thailand. In addition, it matched the 510 bp resolution of Tanaka et al. (2008) only at position G25A. Most haplotypes were only one or two mutation steps apart suggesting recent lineages. We also discovered a specific mutation site (A47G) that was exclusively found in 38 Philippine pigs and was absent in all samples examined, including GenBank samples. Interestingly, three samples clustered under this haplogroup were our samples from F1 hybrids of an endemic Palawan bearded pig (*Sus ahoenobarbus*) from Palawan that was crossed with domestic pigs in captivity under the government breeding and conservation program. The widespread sharing of haplotypes in this haplogroup provided genetic signals that the D7 ancestral lineage covers a wide geographic proximity in different Philippine islands (Figure 1). Here, all samples from the Eastern Visayas region and 11/19 from Palawan were assigned to D7 haplogroup, which may indicate that this haplogroup is associated with Sundaland.

Here we designated European Clade as MC E, forming haplogroups D1 and D4. This result is consistent with previous studies that, unlike MC A, which descended from multiple Asian ancestors, the European wild and commercial breeds descended from a common ancestor, thus forming a monophyletic clade. Haplogroup D1 included all exotic pig breeds together with Northeast Asian domestic and wild boars, which included our two newly sequenced Philippine pig haplotypes (PHL17 and 19). Meanwhile, one haplotype possessed an mtDNA sequence that derived only three mutational distances from wild boar belonging to D6 haplogroup or known as Pacific Clade.

The Lanyu, a unique domestic pig from Taiwan islands, formed an independent clade and was distant from all other pig breeds, but perhaps still belonged to the Asian pig type. Six haplotypes (*n* = 18/184; 9.78%) from our dataset formed a subclade with Lanyu with a significant posterior probability of 97%, which we refer to here as Philippine Lanyu subclade. The presence of three rare, repeated “ACACAAACC” diagnostic motifs in the multiple alignments, a motif possessed by type I Lanyu, and likewise the mutation signatures at positions G90A, C278T, A301G, G534A, A541G, G574A, A657G, and A740T, which correspond to 90, 279, 302, 535, 575, 657, and a transversion at 741 in Li et al. (2017) and 542 in Wu et al. (2007), differed from Asian and European *Sus* progenitors. Although our analysis revealed that the Philippine Lanyu subclade continues to be characterized by a transversion at A143T. It could be speculated that some degree of population subdivision may have occurred due to isolation that is sufficient to warrant recognition of the Philippine Lanyu subclade.

**FIGURE 4:**
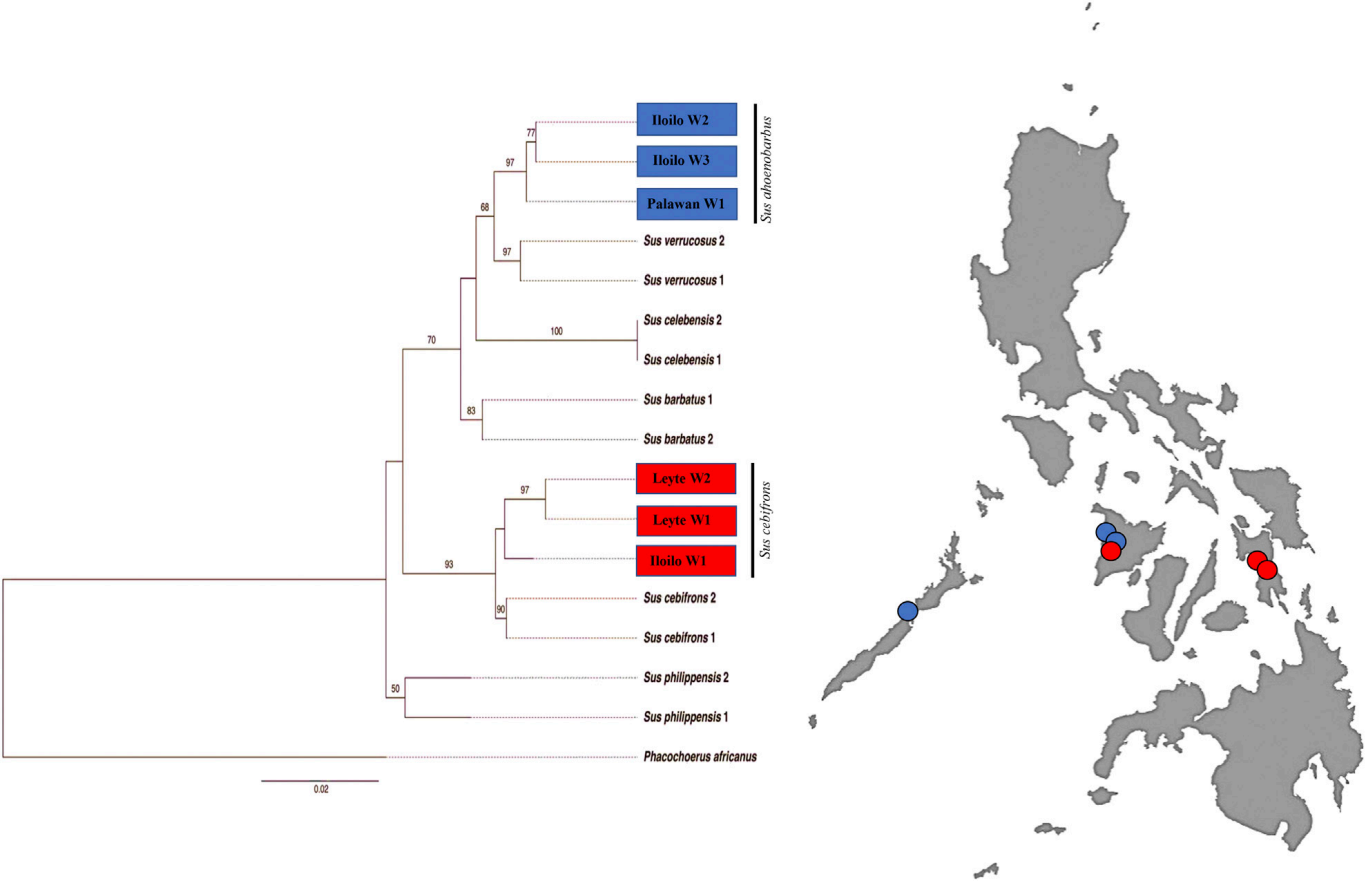
Maximum Likelihood (ML) inference of Philippine wild pigs including the available mtDNA D-loop sequences of wild pigs from the GenBank with GTR + G as the best-fitted model using PhyML ver.3.0 with Warthog (*Phacochoerus africanus*) as outgroup.

To draw further conclusions about the phylogeny of the Philippine wild pigs that fell out of the *S. scrofa* lineage, we analyzed these animals together with the wild pig sequences available in the GenBank database. We found a clear phylogenetic resolution of the relationships between the Philippine wild pigs and the downloaded sequences (Figure 4). For the first time, this study detected an inconsistency in the maternal distribution of the numerous endemic Philippine wild pigs. Haplotype PHL45-46, consisting of wild pigs from Mari-it Wildlife Conservation Park (MWCP), Iloilo and VSU, Leyte, respectively, showed close genetic relationship with *S. cebifrons*. Haplotype PHL47-48, consisting of wild pigs from Palawan and MWCP, showed genetic relatedness to *Sus barbatus*, a subclade of *S. ahoenobarbus* endemic to the Palawan Faunal Region.

**FIGURE 5:**
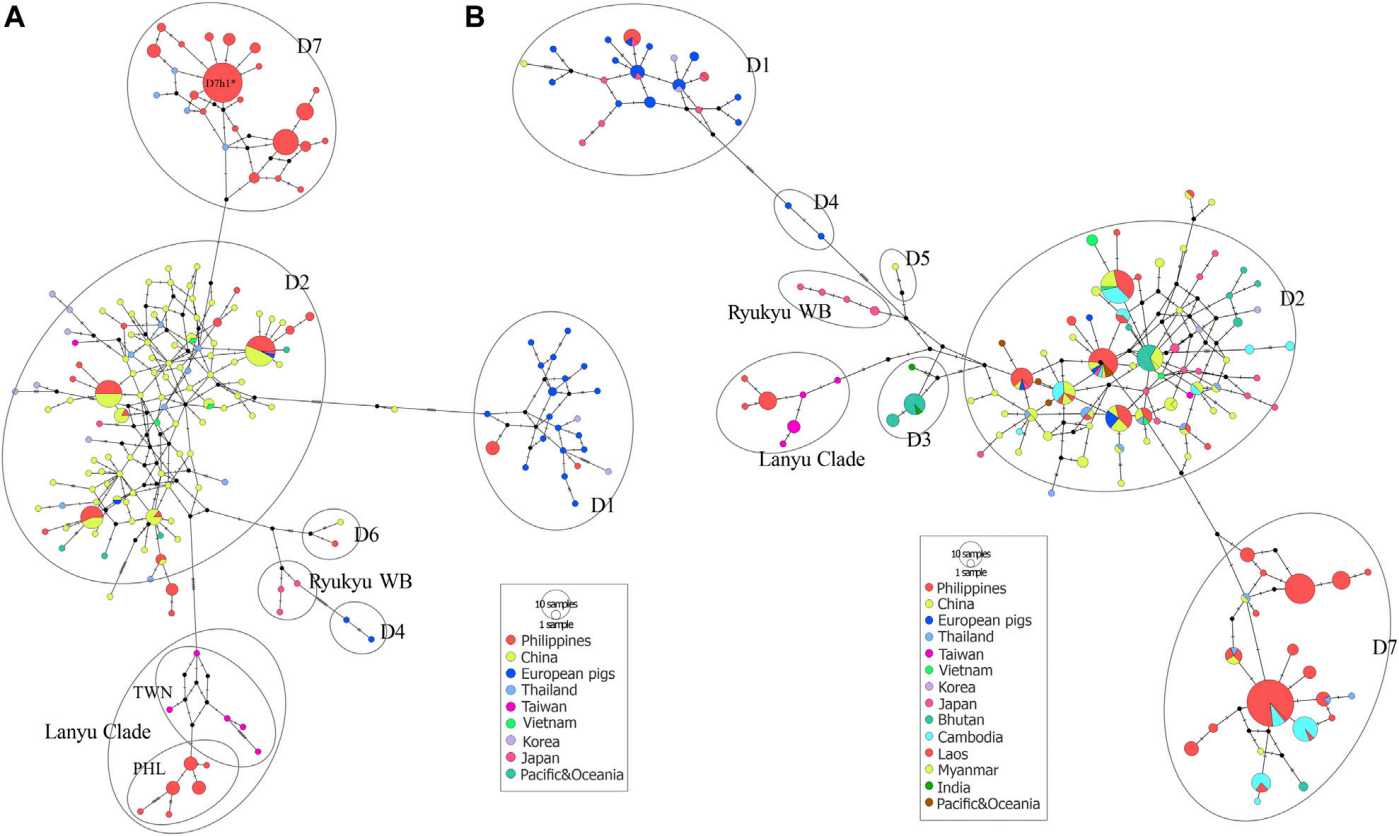
Median-joining network of Philippine pigs **(A)** with global reference sequences of Asian and European pig haplotypes based on complete mtDNA D-loop sequences and a partial MJ network **(B)** using 510 bp to accommodate the major representative haplotypes through the partial mtDNA resolution used in the previous studies for a thorough haplotype representation of the different geographical locations. The size of each circle is proportional to the haplotype frequency. The color represents the regions from which the sequence originated.

The MJ network analyses consistently revealed the independent phylogenetic clusters and clear separation of European pigs from Asian pigs, as well as the Lanyu Clade lineage at least 10 steps away from the macro-Asian group (Figure 5A). Both the phylogenetic networks of the complete (Figure 5A) and partial (Figure 5B) mtDNA D-loop sequences of the Philippine pig together with the global domestic and wild boars showed that they contain founder sources from five different geographic origins, except for the endemic Philippine wild pigs. However, although we identified several Philippine pig lineages, we did not detect D3, D4, and D5 haplotypes in our dataset. The complete mtDNA D-loop sequences showed a concordance of haplotypes clustered together and consistent with their geographic region of origin. Negative correspondence between geographic origin and breeds is shown among individuals from different breeds with shared diverged haplotypes. This was particularly evident in the D2 haplogroup, where the majority of haplotypes were shared transregionally and the observed genetic variation in MJ networks was enormous. This supported the theory of multiple origins of pigs that included present-day China and MSEA (Chittavichai et al., 2021). Compared to Chinese pigs, the degree of European (D1) maternal introgression in Philippine pigs was minimal at 2.86%, although our sampling areas were aggregates of lowland and upland areas. Finally, the overall exponential population growth pattern was evident in the D7 haplogroup, and the high frequency of sequences in D7h1* (potential founder) formed a central node from which the other Philippine pig haplotypes, including the Thai samples, radiated with only one mutational step, consistent with recent population expansion. The clear delineation of the separation of eight Philippine haplotypes by one mutation step (A47G) is also evident.

### Population Expansion and Demographic History

To understand more about the historical background of these populations, we performed a neutrality test to distinguish between neutrally evolving sequences and sequences that evolve under directional selection. Simulations of the neutrality test for the entire dataset yielded high negative values and a significant Fu’s F_*S*_ test indicated possible population expansion in the past (Table 2). This supported the MJ network, as several haplotypes appeared to harbor an excess of rare singletons beyond that expected under neutrality. Similarly, the two major haplogroups D2 and D7 showed negative and highly significant (*p* < 0.01) Tajima’s *D* and Fu’s F_*S*_ test values.

**FIGURE 6:**
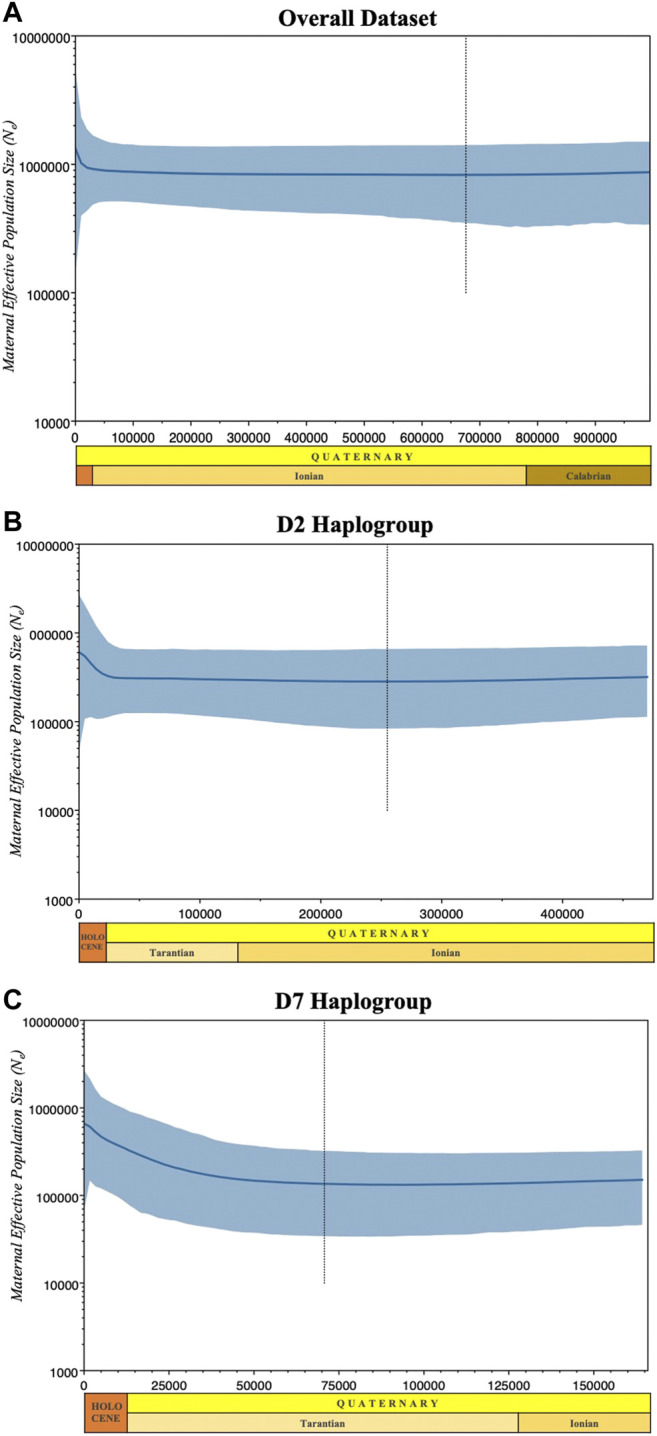
Bayesian skyline plots showing the effective population sizes of the overall dataset **(A)**, the predominant **(B)** D2 and **(C)** D7 haplogroups. Median estimates of female effective population size (*N*_*e*_) are shown as a solid thick line (blue) and the light blue shaded area marks the 95% credibility intervals. The abscissa is scaled in thousands of years before the present (BP).

We also evaluated the changes in effective maternal population sizes with BSP based on coalescent theory for the overall dataset (Figure 6A) and the predominant haplogroups D2 (Figure 6B) and D7 (Figure 6C). Consistent with the population expansion hypothesis, BSP projected an increase in effective population size for the D7 haplogroup, with an imminent population increase occurring around the interglacial periods of the Late Pleistocene. The D2 haplogroup showed a slight increase in effective population size at about 25,000 YBP. Overall, the statistics of the neutrality test and the past population dynamics of Philippine pigs suggest a possible population expansion of domestic pigs in the Philippines prior to the possible initial domestication of the wild boar *S. scrofa*.

## Discussion

This study is the first comprehensive screening of the complete mtDNA D-loop variation of Philippine pigs to clarify their past dispersal history by performing phylogenetic analysis together with domestic pigs and wild boars representing Asian and European pigs. Due to limited molecular studies and lack of archaeological evidence supporting the domestication of pigs in the Philippines, there has long been a controversy over the absolute conclusion that Philippine pigs descended from Philippine wild boar ancestors. In this study, the context of genetic diversity, phylogeography, population dynamics, and extent of genetic introgression of Philippine pigs were inferred together with the domestic pigs and wild boars that roughly corresponded to their geographic origin. Based on the patterns of mtDNA D-loop variation in Philippine pigs, our results were consistent with a clear phylogenetic pattern showing two core lineages of *S. scrofa*, both of Asian and European phylogeographic origin, occurring in the Philippine pig population. The enormous genetic variation in Asian pigs and the frequent overlap of haplotypes among Asian pigs were also noted, especially among the Chinese and MSEA domestic and wild boars in the Philippine pigs, accounting for 93.48% of the studied populations. Therefore, this wide distribution of haplotypes derived from the Eurasian continent present across the Philippine islands may indicate a genetic signal that could corroborate a gene flow that may have resulted from human migration and trade. Recently, there have been reports of at least five waves of human migration into the Philippines (Larena et al., 2021). This phenomenon may have paved the way for the introduction of domestic pigs into the Philippines with multiple lineages, including domestic animals such as chickens (Thomson et al., 2014), goats (Naderi et al., 2007), cattle (Scott, 1990), and other species that have adapted to local conditions and developed distinctive traits.

**FIGURE 7:**
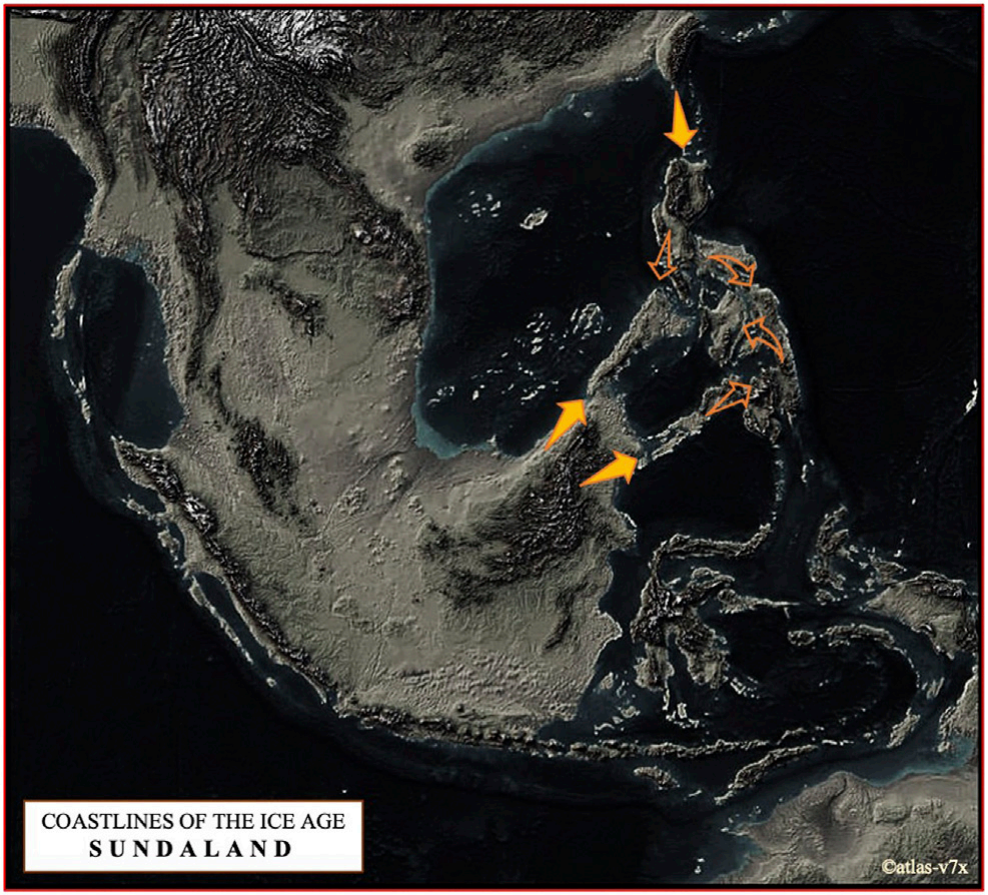
The map shows a terrain that may have been formed during the Last Glacial Maximum about 21,000 years ago, when sea level was about 125 m below the present level (©atlas-v7x). We have proposed here two dispersal routes of pigs to the Philippine Archipelago, one via NEA via Taiwan in parallel with the Neolithic expansion in ISEA and Oceania, and the other via Southeast Asia, especially from the Indochinese Peninsula via Sundaic Region to Palawan and the Sulu Archipelago.

Based on the complete mtDNA D-loop sequences, we propose that the pattern of current maternal haplotype distribution of Philippine pigs is derived from populations descended from the predominantly diverse domestic pigs and wild boars of the Eurasian continent that entered the Philippine archipelago via two routes (Figure 7). One is *via* NEA through Taiwan, in parallel with the Neolithic expansion into ISEA and Oceania, and the other is via Southeast Asia, particularly from the Indochinese Peninsula via the Sundaic Region (Sundaland) to Palawan and Sulu Archipelago, which spread to the rest of the Philippine islands since prehistoric times. The latter could be consistent with the previously suggested north to south dispersal pattern (Koenigswald, 1956; Arenas et al., 2020; Antoine et al., 2021), while the former might agree the dispersal routes proposed by (Porr et al., 2012; Mijares, 2014; Louys et al., 2018; Arenas et al., 2020). Despite geographic barriers to migration, these animals have been able to expand their range across the various islands of the Philippines, and variation in morphological patterns has evolved among these populations. These patterns of genetic variation may also reflect the multifaceted history of rich trade and barter between travelers and coastal communities, including river movements in coastal settlements in the Philippines in prehistoric and protohistoric times (Fox, 1967). For example, the extent of trade networks around the South China Sea and the Austronesian trade sphere, which included MSEA, Indonesia, the Philippines, Taiwan and southern China, and India to the west (Hung et al., 2007; Alam et al., 2021) is very complex, which could likely be linked to the enormous movements of domestic animals and other material cultures. This could include domestic pigs, where episodic admixture of pig lineages from different geographic regions may have occurred, as indicated by the mtDNA signatures of present-day Philippine pig populations. Although some suggest advanced navigation techniques would be needed to connect distant islands (Arenas et al., 2020), archaic fossil suggest that humans have started navigating around 60,000 years ago (Stringer, 2000; Balme, 2013; Malaspinas et al., 2016; Norman et al., 2018; Arenas et al., 2020) that may have indeed initiated these processes. This hypothesis was further supported by contemporary mtDNA studies as earlier revealed the expansion of modern humans that has occurred through long-distance dispersal events (Arenas et al., 2020). Our result also supports the hypothesis that even before the arrival of Europeans in the Philippines, pigs were already introduced by Chinese traders before the later importation of various exotic European pig breeds (Bondoc, 2008). This hypothesis is evident precisely in the close genetic relationship between Philippine and Chinese pigs (referred herein as the D2 haplogroup), as evidenced by the similarities in their morphology due to genetic introgression (Figure 2), which also led to the myriad phenotypic differences between these native pig populations. Rigorous inter-island transport was also well observed, resulting in genetic admixture between these populations as part of the valuable resource for economic trade and cultural exchange in the Philippines. Although our samples were from both lowland and upland areas, the extent of European maternal introgression in Philippine pigs was minimal at 2.86% compared to pigs of Asian ancestry. This could indicate that the exotic pig breeds have not yet penetrated the remote areas of the country. Likewise, this reflects that most farmers prefer indigenous pigs over exotic sows because they are more vigorous and adaptable to adverse environmental conditions, and resistant to pests and diseases.

For the first time this study provides evidence for the presence of the Philippine Lanyu subclade with a relatively high haplotype frequency compared to previous records. This even surpasses the haplotypes identified in Taiwan (Li et al., 2017), where these rare pigs are thought to have originated. Demographic signals were also indicative that genetic exchange of these rare pigs with other domestic pigs in the Philippines may have been existed for some time, in contrast to the reported scenario in Taiwan where increasing inbreeding within the small Lanyu population has become a conservation concern (Chang et al., 2009). The Lanyu pig has only been documented at Lanyu Islet off the coast of Taiwan (Cheng, 1986) and is generally absent from the Eurasian continent and other neighboring islands. It is one of several breeds whose domestication has been described as cryptic (Larson et al., 2010) as they are morphologically distinct from other Chinese pig breeds (Luetkemeier et al., 2010). Considering the lack of a clear genetic source and its limited distribution, even during periods of low sea level in the Pleistocene when land bridges connected the islands of Japan, Ryukyus, Lanyu and Taiwan (Larson et al., 2010), it could be assumed that rapid dispersal did not occur resulting in a reduction in diversity. In contrast to Philippine Lanyu subclade, and despite the geographic location of the Philippines, pigs carrying these genetic signatures have persisted on multiple islands and dispersed in the Northern Luzon range toward the western and central regions of the Philippines. We hypothesized that the rate of genetic divergence may have been accelerated due to the smaller size of the ancestral population, unlike other haplogroups with larger population sizes and ranges, e.g., D2 and D7, which precluded divergence into an independent evolutionary entity. Despite the absence of historical records of geographic contact between the Philippines and Taiwan, the migration of these pigs may have been assisted by humans (Chittavichai et al., 2021), which may be consistent with the presumed movements of Austronesians from Taiwan to the Philippines about 3,000 years ago (Bellwood, 2005). However, the absence of domestic pigs in Taiwan at a similarly early date has led scholars to question the possible Neolithic introduction of domestic pigs to the Philippines (Li et al., 2017). Similarly, it contradicts the results of ancient DNA and morphometric studies of modern and archaeological pigs from ISEA (Dobney et al., 2008). Currently, the hypothesis of whether domestic pigs existed in Neolithic Taiwan has not been resolved (Chuang, 2021). Although various literature indicated that the Neolithic expansion was associated with the movements of domestic animals (Diamond and Bellwood, 2003; Piper, 2017), a recent study suggest that this was unlikely for chickens as it favored the translocation route from MSEA *via* Sundaland, and subsequently followed by the southward diffusion from the Philippines into the Pacific islands (Godinez et al., 2021). Moreover, contrary to the prediction of the out-of-Taiwan theory, there is recent evidence of gene flow of indigenous rice from Northern Luzon to Taiwan that occurred ∼1,300 years ago (Alam et al., 2021) favoring the hypothesis of south to north expansion. Thus, this finding may stimulate scientific interest in the complexity of the introduction and dispersal of domestic pigs, particularly the Lanyu pig into the Philippines. To date, we have found no clear genetic evidence that the Lanyu pig was likely first domesticated in the Philippines and brought to Taiwan at some point in the past, subjected to human-assisted dispersal or likely experiencing vicariance from Taiwanese populations.

Unlike other *Sus* lineages, very little is known about the distribution of haplogroup D7, i.e., it is absent from the Insular and NEA regions. Therefore, a sufficient and complete description of the mtDNA D-loop fragments might be plausible in formulating a robust hypothesis to explain distribution and demography. In our results, we identified 20 unique haplotypes of D7 that occurred in several major Philippine Islands (Palawan, Cebu, Samar, Panay, Guimaras, and Bohol), were morphologically variable (Figure 2), and had significantly larger ancestral population sizes compared to all other identified *Sus* lineages. These are the highest haplotypes that represent the first complete mtDNA D-loop fragments reported from this haplogroup to date. Although few molecular studies have clarified the diversity and genetic characteristics of pigs from the Sundaland and given the absence of a similar haplotype in Northern Philippines, we propose that the ancestral population of this haplogroup likely dispersed to the Philippines from MSEA via the Sunda region (which was merged with the Asian continent during the Pleistocene) through Sulu Archipelago off the coast of Mindanao Islands and Palawan and spread throughout the Philippine islands. Looking at the biological components of these regions, the species diversity of mammals found in Eastern Visayas is little different from that found in the same habitat in Sulu Archipelago, Bohol, and the Mindanao archipelago. This is probably because during the recent Ice Age, Eastern Visayas, including Bohol Island was comprised the “Greater Mindanao Faunal Region” connected by land bridges during the Pleistocene (Oliver and Heaney, 2008) allowing animals to translocate and disperse. While the fauna of Palawan is more similar to that of Borneo (Heaney, 2002), this route could be the main route (including Sulu Archipelago) for the immigration of pigs from Borneo to the Philippines. Hence, the presence of more than one lineage of porcine mtDNA has been detected in Palawan alone, including the rare Lanyu pigs. This dispersal scenario could also be consistent with the D6 haplotype found in Bohol Island. The ongoing debate over the contradicting hypothesis of Larson et al. (2005) that ISEA (Yang et al., 2011) rather than MSEA is the center of domestication of the so-called Pacific Clade has resurfaced in recent years. Although we acknowledge the possibility that this clade was domesticated in eastern Indonesia (Yang et al., 2011) and transported back to MSEA, we support the claim of Larson and coworkers because the abundance of wild boars in MSEA, and part of South China may strongly support the inference that MSEA is the center of domestication of this haplogroup. We speculated that the present-day D6 haplotypes in ISEA may simply be a product of demographic expansion influenced by human-mediated dispersal (i.e., the association of Neolithic material culture between Vietnam and ISEA), as previously highlighted by Gongora et al. (2004) as a major driving factor that established the present-day geographic distribution of *Sus* populations around the world. Due to long-term gene flow within and between wild and native species, and subsequent intensive breeding practices in recent centuries, modern populations that bear ambiguous resemblance to their early ancestors have emerged. Thus, such a phenomenon has resulted in a gene flow pattern that often produces modern populations that appear to have originated outside the region of original domestication (Larson and Burger, 2013). Therefore, such an episode could not be used to support the occurrence of major domestication of this haplogroup, and careful consideration should be taken to avoid similar circumstances in the future, especially when limited genomic markers are involved.

The signature of population expansion was evident in the neutrality test statistics (Table 2). The Fu’s F_*S*_ test, based on haplotype frequencies, and Tajima’s *D* test, based on the difference between the number of polymorphic sites and the mean number of pairwise nucleotide differences, indicate an expanding population in two major haplogroups (D2 & D7) and the entire datasets. This positive sign of population expansion was evident in the star-like pattern in the MJ network, large negative values and highly significant (*p* < 0.01) values of the neutrality test (Table 2), a feature that is a signature of population expansion. Based on the estimation of pairwise divergence of populations, the low genetic differentiation of populations from Eastern to Central regions could precede Southern regions as the likely origin of eastward expansion and dispersal into the Central Regions of the Philippines. This could correlate with the northwestward gene flow of Mamanwa Negritos into the Philippines that occurred via Mindanao, probably via the Sulu Archipelago (Larena et al., 2021).

### Disparity Between Mitochondrial DNA and the Distribution Range of Endemic Philippine Wild Pigs, and Its Conservation Initiatives

The results of the patterns of mtDNA variation showed that Philippine pigs are not maternally descended from Philippine wild pigs, contradicting the earlier information that they are maternally descended from numerous endemic wild pigs in the country (Eusebio, 1969). While our study only limits the evidence of maternal inheritance, we cannot rule out the possibility of paternal admixture of these wild pigs into the native pig populations, as hybridization with a male wild pig is a common practice among most farmers. Therefore, it is imperative that studies using Y-specific markers be conducted to clarify the extent of male-mediated introgression of Philippine wild pigs into native pig populations. Unlike the wild boar *S. scrofa*, which is ubiquitously distributed throughout the Philippine archipelago, Philippine wild pigs are biogeographically isolated from each other at different Faunal Regions (Oliver, 1995; Ingicco et al., 2017) (see more on Supplementary Table S3A). We believe that the extensive domestication episode of these wild pigs has not progressed, whereas this may have been the case for some other Southeast Asian wild pigs such as *S. celebensis* (Oliver and Birsbin, 1993). Due to an alarming population decline, it has recently been classified as a Critically Endangered species by the International Union for Conservation of Nature Red List (IUCN, 2016), mainly due to intensive hunting and loss of forest habitat. Therefore, the pressure to adopt conservation measures has become challenging over the years such that the government has taken initiatives like captive research and development based on win-win conservation measures (Linkie et al., 2015) to save these wild pigs from extinction. Currently, protected areas (e.g., parks) as well as breeding centers are strategically located in faunal regions where these wild pigs are geographically distributed such as in Palawan, Negros, Panay, Leyte, and Luzon. As part of these conservation initiatives, our sample includes an F1 hybrid *S. ahoenobarbus* crossed with native pigs from Palawan carrying a maternal lineage of *S. scrofa* of haplogroup D7. Visual observations of these animals revealed variations in morphology among the offspring (i.e., the color pattern of bristles and hairs on the head) due to the different breeding practices. These pronounced morphological variations are due to the fact that mtDNA is maternally inherited and breeding between a male wild pig and a female native pig is often preferred by farmers for behavioral and physical reasons.

Hints of discrepancy between the molecular data, distribution, and observed morphological representation of these four endemic wild pigs have been observed in this study. For example, for *S. cebifrons*, which is reported only in the “Greater Panay-Negros Faunal Region” (GPNFR - Panay, Negros, Guimaras, Cebu, and Masbate Islands; Oliver and Heaney, 2008), the presence of mtDNA footprints was detected in Palawan (Greater Palawan Faunal Region; GPFR). Similarly, the GPNFR shows a genetic presence of *S. ahoenobarbus*, which is endemic to the GPFR. Thus, this is the first record of *S. ahoenobarbus* and *S. cebifrons* in GPFR and GPNRF, respectively. Since its last documentation (Oliver and Birsbin, 1993), their range is consistent with the expected distribution pattern and there has been no evidence of mixing of species in the past (Oliver, 1995). We suggest that an altered distribution pattern already existed in these contemporary Philippine wild pigs or that it was overlooked by previous researchers. Our study therefore suggests that contemporary morphology should be re-evaluated, including the molecular aspect, to shed more light on the complexity of the distribution pattern and variation of these interesting animals.

## Conclusion

Analysis of mtDNA D-loop sequences from Philippine pigs has contributed significantly towards completing the sparse molecular studies on the evolutionary history and biogeography of pigs in the Philippines. We have uncovered the close genetic linkage between continental wild boars and domestic pigs originating from the MSEA and NEA regions present in the Philippine pig genetic pool, which may have resulted from several waves of human migration and trade in the Philippines. Two possible routes of dispersal are suggested. One leads through Northeast Asia regions that paralleled the Neolithic expansion in ISEA and Oceania, and the other leads from MSEA that may have passed through the Sundaic region to Palawan and the Sulu Archipelago since prehistoric times. The signals of inconsistency between the maternal pattern, morphology, and range of the numerous wild pigs open a new challenging approach to elucidate the complexity of these interesting animals. Thus, conservation initiatives based on win-win conservation measures should be a priority.

## Data Availability

The datasets presented in this study can be found in online repositories. The names of the repository/repositories and accession number(s) can be found in the article Supplementary Material.
