# Supplementary figures and images for: Origin and Demographic History of Philippine Pigs Inferred from Mitochondrial DNA
Source: Front Genet. 2022 Jan 25;12:823364. doi: 10.3389/fgene.2021.823364 (PMC8822243; doi:10.3389/fgene.2021.823364)

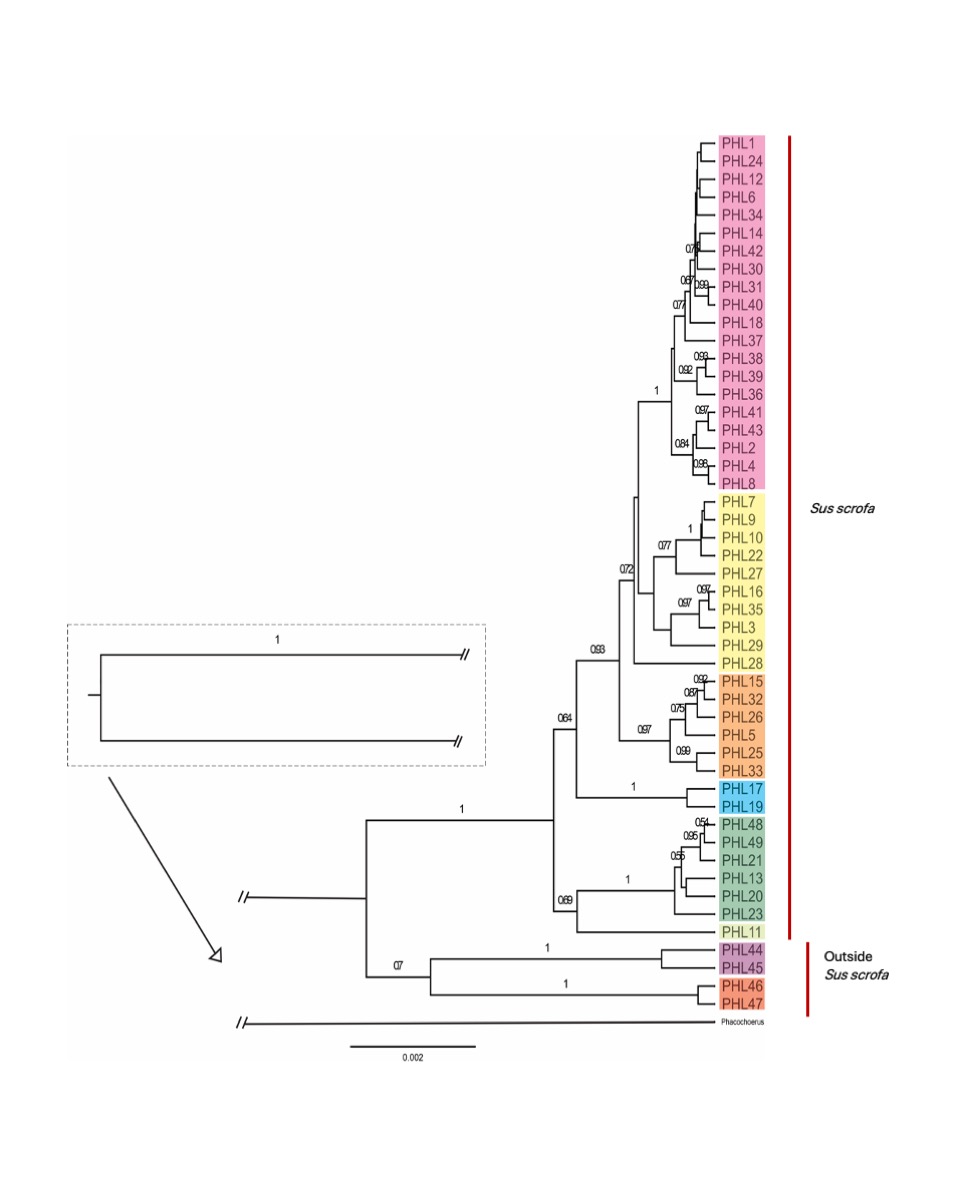

Supplement: Supplementary file 1 [file Image1.JPEG]

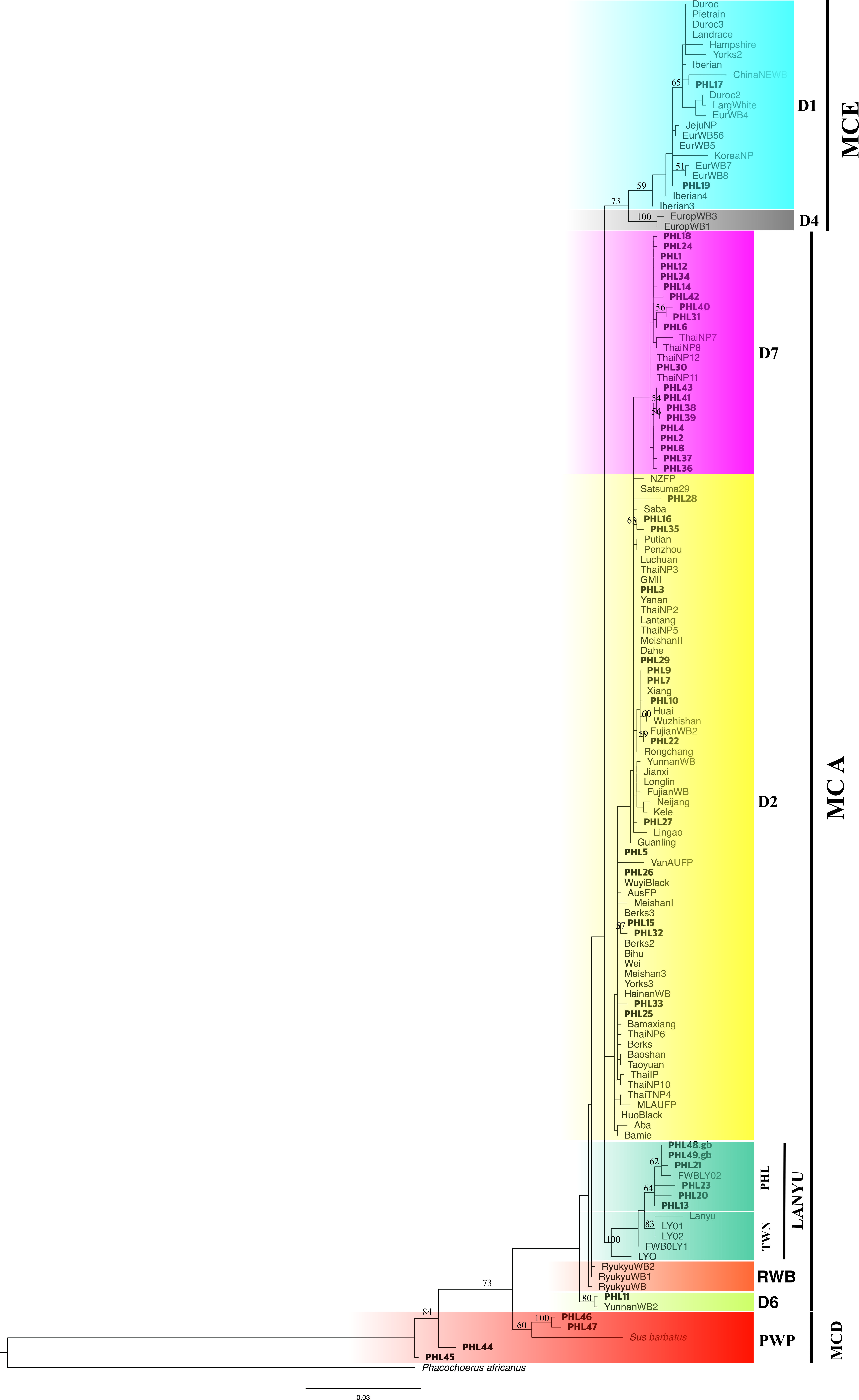

Supplement: Supplementary file 2 [file Image2.JPEG]
